# Chronic Stress Induces Structural Alterations in Splenic Lymphoid Tissue That Are Associated with Changes in Corticosterone Levels in Wistar-Kyoto Rats

**DOI:** 10.1155/2013/868742

**Published:** 2013-02-10

**Authors:** María Eugenia Hernandez, Lucia Martinez-Mota, Citlaltepetl Salinas, Ricardo Marquez-Velasco, Nancy G. Hernandez-Chan, Jorge Morales-Montor, Mayra Pérez-Tapia, María L. Streber, Ivonne Granados-Camacho, Enrique Becerril, Baquera-Heredia Javier, Lenin Pavón

**Affiliations:** ^1^Laboratorio de Psicoinmunología, Dirección de Investigaciones en Neurociencias, Instituto Nacional de Psiquiatría Ramón de la Fuente, 14370 Tlalpan, DF, Mexico; ^2^Laboratorio de Farmacología Conductual, Dirección de Investigaciones en Neurociencias, Instituto Nacional de Psiquiatría Ramón de la Fuente, 14370 Tlalpan, DF, Mexico; ^3^Department of Neuropathology, National Institute of Neurology and Neurosurgery, 14269 Mexico City, DF, Mexico; ^4^Departamento de Inmunología, Instituto Nacional de Cardiología, 14080 México, DF, Mexico; ^5^Departamento de Inmunología, Instituto de Investigaciones Biomédicas, Universidad Nacional Autónoma de México, 70228 México, DF, Mexico; ^6^Departamento de Inmunología de la Escuela Nacional de Ciencias Biológicas, Instituto Politécnico Nacional, 11340 México, DF, Mexico; ^7^Departamento de Investigación Experimental y Bioterio, Instituto Nacional de Ciencias Médicas y Nutrición, 14000 México, DF, Mexico; ^8^Laboratory of Surgical Pathology, The American British Cowdray Medical Center, 01120 Mexico City, DF, Mexico

## Abstract

Major depressive disorder patients present chronic stress and decreased immunity. The Wistar-Kyoto rat (WKY) is a strain in which the hypothalamic-pituitary-adrenal axis is overactivated. To determine whether chronic stress induces changes in corticosterone levels and splenic lymphoid tissue, 9-week-old male rats were subject to restraint stress (3 h daily), chemical stress (hydrocortisone treatment, 50 mg/Kg weight), mixed stress (restraint plus hydrocortisone), or control treatment (without stress) for 1, 4, and 7 weeks. The serum corticosterone levels by RIA and spleens morphology were analyzed. Corticosterone levels as did the structure, size of the follicles and morphology of the parenchyma (increase in red pulp) in the spleen, varied depending on time and type of stressor. These changes indicate that chronic stress alters the immune response in the spleen in WKY rats by inducing morphological changes, explaining in part the impaired immunity that develops in organisms that are exposed to chronic stress.

## 1. Introduction

Chronic stress induces immunosuppressive effects and increases susceptibility to various diseases, such as infection, autoimmune disease, and neoplasia [1]. In addition, clinical studies have shown that patients with psychiatric disorders, such as major depression, develop symptoms of chronic stress and mount attenuated immune responses [2].

To study the function of stress in depression, several animal models have been developed, permitting researchers to examine aspects of neurobiology of this disease. One of the most important systems that controlled the stress response is the hypothalamus-pituitary-adrenal axis (HPA), or stress axis. Wistar-Kyoto rats (WKY) are hypersensitive to stress and display dysregulation of the HPA axis, in association with anhedonic behavior [3]. The principal mediators of the stress axis are corticotropin-releasing hormone (CRH) in the paraventricular nucleus of the hypothalamus, adrenocorticotropin (ACTH) in the anterior pituitary gland, and glucocorticoids (corticosterone in rodents and cortisol in humans) in the adrenal glands.

To maintain homeostasis, glucocorticoids establish a negative feedback loop through specific receptors [4]. However, when the HPA axis is activated repeatedly or chronically, such as during chronic or excessive stress, an allostatic load is generated, which can alter its integrators (nervous neurotransmitters [5], endocrine hormones [6], and immune system secondary lymphoid organs [7]).

During chronic stress, glucocorticoid levels rise significantly and induce a negative immune response [8]. Clinical studies have reported that depressed patients have high cortisol levels [9, 10]. However, there is no direct evidence of this effect on secondary lymphoid organs or physical health in depressed subjects. Thus, the aim of our study was to determine the effects of various stressors (restraint, hydrocortisone, and mixed treatment), applied chronically, on corticosterone levels and the cytoarchitecture of splenic lymphoid tissues in WKY rats.

## 2. Materials and Methods

### 2.1. Animals and Stress Exposure

Male Wistar-Kyoto rats (180–200 g) were purchased from Harlan (Indianapolis, IN, USA) and group-housed in clear plastic containers (450 × 240 × 200 mm) with food and water *ad libitum *under 12:12 h light/dark cycles. Animals were maintained at 23°C and underwent one week of habituation before the experiments were begun. All procedures were performed per the Mexican legislation (NOM-062-ZOO-1999; SAGARPA), based on the Guide for the Care and Use of Laboratory Animals, NRC. The INPRF Committee for Animal Care and Use (IACUC) approved and supervised our experiments.

WKY rats were assigned to experimental groups (*n* = 6): (a) restraint stress (IMM); (b) hydrocortisone (HC); (c) restraint stress and hydrocortisone (IMM + HC)—that is, mixed; (d) control. In Group A, the stress was generated by restraining the rats in an adjustable cylindrical plastic tube (250 mm long × 75 mm diameter) for 3 h once daily. Group B received hydrocortisone (50 mg/kg weight) in their drinking water daily. In Group C, stressed rats were fed hydrocortisone. All groups were stressed for one, four, and seven weeks. Group D comprised untreated control rats.

### 2.2. Preparation of Hydrocortisone

Hydrocortisone (50 mg/kg), prepared daily, was dissolved in ethanol (2.2 p. 100) and added to drinking water.

### 2.3. Measurement of Plasma Corticosterone (CORT)

Rats were sacrificed by rapid decapitation in the morning (10:00 h) or immediately after the restraint stress test. Trunk blood was collected into Vacutainer tubes and centrifuged at 2500 rpm for 15 min at 4°C, and the serum was stored at −70°C until analysis. Serum CORT was analyzed using an RIA kit (Siemens Medical Solutions Diagnostics, Los Angeles, CA, USA). The Coat-A-Count Rat Corticosterone method is a solid-phase ^125^I-radioimmunoassay, the detection limit of which is 5.7 ng/mL. The samples (50 *μ*L) were assayed in duplicate per the manufacturer's instructions, as described. The intra- and interassay variability (% CV) was <8 p. 100 and 9 p. 100, respectively.

### 2.4. Preparation of Tissue for Histological Examination

Spleens were fixed in paraformaldehyde 4 p. 100 for 24 h, paraffin embedded, sectioned serially (4 to 5 *μ*m slices), and stained with hematoxylin and eosin.

### 2.5. Statistical Analysis

Data are presented as mean ± SD, calculated from each experimental group (six rats per group). Statistical significance was determined by one-way ANOVA, followed by Bonferroni's *post hoc* test for comparison. *P *values of less than 0.05 were considered statistically significant.

## 3. Results

### 3.1. Body and Adrenal Gland Weight

**Figure 1:**
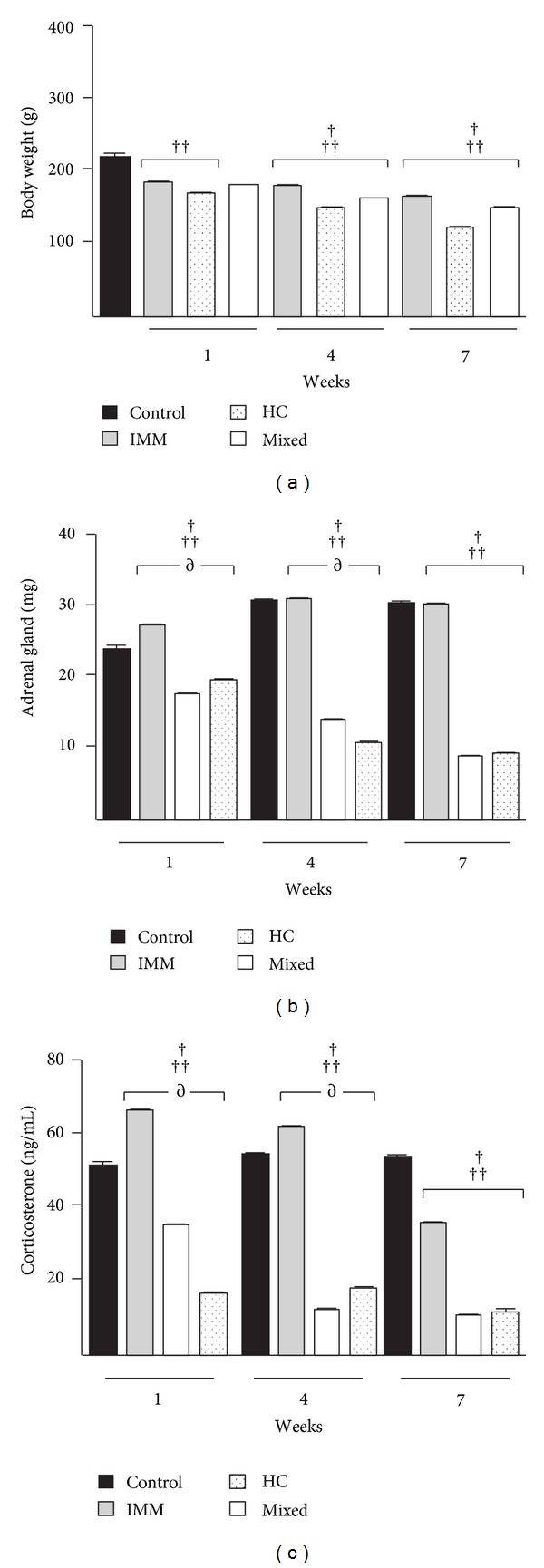
Effect of stressors on body and adrenal gland weight and corticosterone levels in Wistar-Kyoto rats. Wistar-Kyoto rats (*n* = 6; per group) were treated with restraint stress (immobilization for 3 h daily) or hydrocortisone at 50 mg/kg or restraint stress plus hydrocortisone (mixed group) for 1, 4, and 7 weeks. (a) Body weight, (b) adrenal gland weight, and (c) corticosterone levels. Experimental groups showed significant differences compared to the control group at 1, 4, and 7 weeks of treatment. ^†^ *P* < 0.001 between immobilization and hydrocortisone groups; ^††^ *P* < 0.001 between immobilization and mixed groups; ^∂^ *P* < 0.001 between mixed and hydrocortisone groups. Data are expressed as mean ± SE.

Figures 1(a) and 1(b) show the body and adrenal gland weight of rats by treatment (C, IMM, HC, and mixed). Body weight fell in all groups (F_(9,50)_ = 93.16, *P* = 0.001) compared to control group at one (C = 218 ± 13 versus IMM = 189 ± 4; HC = 179 ± 3; mixed = 169 ± 6), four (C = 218 ± 13 versus IMM = 179 ± 9; HC = 162 ± 4; mixed = 150 ± 4), and seven (C = 218 ± 13 versus IMM = 169 ± 5; HC = 151 ± 3; mixed = 125 ± 5) weeks of treatment. The comparison between treatments by Bonferroni's *post hoc* test showed statistical significance (IMM versus Mixed groups at 1 week); (IMM versus HC and Mixed groups at four weeks); (IMM versus HC and Mixed groups at seven weeks). In contrast, adrenal glands increased in weight in IMM group compared to control group (C = 24 ± 1 versus IMM = 27 ± 0.70) at one week of restraint and remains during the others weeks. Moreover, weight declined after one (HC = 18 ± 0.7; Mixed = 20 ± 0.2), four (C = 31 ± 0.6; HC = 14 ± 0.6; Mixed = 11 ± 0.46), and seven (C = 30 ± 0.3; HC = 9 ± 0.3; Mixed = 9 ± 0.2) weeks of the other treatment. Adrenal glands showed significant effect (F_(11,60)_ = 1170, *P* = 0.001) and comparison between experimental groups showed statistical significance (IMM versus HC and Mixed groups at one and four weeks) and (IMM versus HC and Mixed groups at seven weeks).

### 3.2. Corticosterone Levels

Corticosterone levels (Figure 1(c)) showed contrast and significant changes (F_(11,60)_ = 716.6, *P* = 0.0001). While IMM group showed increased levels (C = 52 ± 3 versus IMM = 67 ± 2); (C = 55 ± 3 versus IMM = 63 ± 2) after one and four weeks, respectively, HC and mixed groups showed reduced levels along the followup in all weeks (C = 52 ± 3 versus HC = 36 ± 1.5 and Mixed = 18 ± 2.5; 1 week), (C = 55 ± 3 versus HC = 13 ± 1 and Mixed = 19 ± 1.5; 4 weeks) and (C = 54 ± 2 versus HC = 12 ± 0.4 and Mixed = 13 ± 2; 7 weeks). The comparison between treatments showed statistical significance (IMM versus HC and Mixed groups; Mixed versus HC group at 1 week); (IMM versus HC and Mixed groups; Mixed versus HC group at four weeks); (IMM versus HC and Mixed groups at seven weeks).

### 3.3. Histological Analysis of the Spleen

#### 3.3.1. Control Group

**Figure 2:**
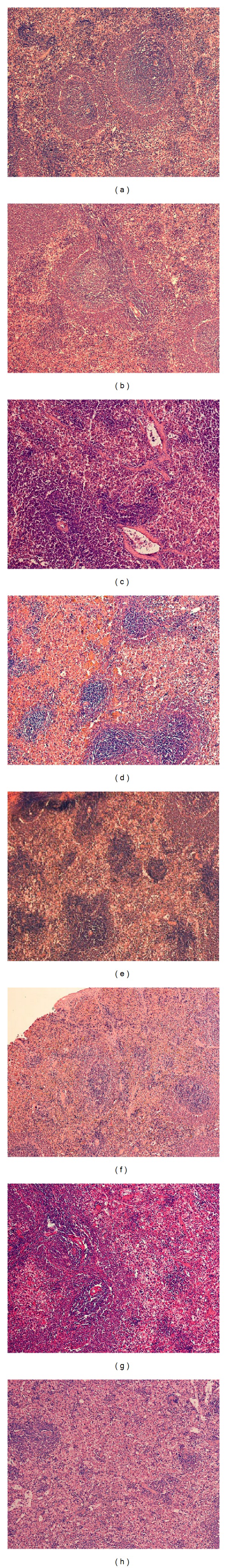
Photomicrographs of spleen from Wistar-Kyoto rats: (a and b) without treatment; (c) treated with restraint stress for 1 week; (d) after 7 weeks of immobilization; (e) after 1 week with hydrocortisone; (f) after 7 weeks of hydrocortisone; (g) after 1 and 7 weeks of mixed treatment (hematoxylin and eosin stain; magnification, 100x).

The histological architecture of the spleen was preserved along the experimental followup. White pulp was seen as large secondary follicles with an image of a “double halo” due to the prominence of the marginal zone. It comprised an assemblage of two vaguely delimited round or oval structures that were superimposed, with an axial profile of the penicillated artery in the center of the inner structure. Each layer contained a mixture of cells, primarily small lymphocytes without any evidence of nuclear atypia or activation (mitotic/apoptotic activity, clearing of chromatin-prominent nucleoli, and macrophages with cytoplasmic tangible bodies) (Figures 2(a) and 2(b)).

#### 3.3.2. Immobilization Group

Notably, in the immobilization group, there was an expansion of the red pulp compartment and involution of the white pulp, with loss of the prominent marginal zone. In contrast, at seven weeks, the red pulp continued to expand, without significant changes in composition, at the expense of the surface that was occupied by the white pulp (Figure 2(d)).

#### 3.3.3. Hydrocortisone Group

At one week, the hydrocortisone group had essentially the same histological appearance as the control group. The cellular composition of the red and white pulp did not vary. There was no evidence of hematopoietic activity in this microscopic field (Figure 2(e)). However, at week seven, compared with the control group, there was a reduction in the proportion of follicular structures; thus, the ratio of red pulp and marginal zone lost (Figure 2(f)).

#### 3.3.4. Mixed Group

Versus the control group, the rats that received mixed treatment experienced a loss of white pulp and a decrease in the size of the follicles and the marginal zone. The greatest difference between groups occurred by seven weeks, wherein the pulp was distorted, favoring the red pulp in all groups. The surface ratio favored the red pulp nearly to 4 : 1 in the mixed group, maintaining the spacing of the tissue elements (Figure 2(h)).

Despite the subtle changes between immobilization- and hydrocortisone-treated groups, we noted a consistent trend in the disarray of the pulp. The mixed treatment group experienced more significant alterations, favoring an increase in red pulp and in the structure and size of the follicles. After four weeks of stress, there were no histological differences compared with that at one week.

## 4. Discussion

Glucocorticoids are characteristic mediators of stress responses, and the detrimental effects of chronic stress on health have been proposed to be attributed to their immunosuppressive properties [11]. Thus, we examined the effects of stressors (immobilization, hydrocortisone, and their combination) on corticosterone levels in WKY rats and determined whether they had adverse long-term effects on splenic morphology.

We observed that all experimental groups display a decrease in body and adrenal gland weight, associated with a decline in corticosterone levels; the least extensive changes were observed in the group of immobilization after one week. Notably, the magnitude of these decreases was dependent on the stressor (mixed > hydrocortisone > immobilization). These results are consistent with other animal studies that have demonstrated that repeat restraint and exogenous corticosterone lower body and adrenal gland weight [12, 13].

The mixed treatment lowered all parameters significantly; we expected that the two component stressors would synergize. The significant reduction in corticosterone levels might be attributed to the length (days to weeks) and extent of the stressor, which should have desensitized the HPA axis-desensitization that might have been caused by impaired glucocorticoid receptor expression and function [14]. Something similar is observed in major depression patients when they receive the dexamethasone suppression test (DST) [15].

Few studies have investigated the effects of chronic stressors on lymphoid organs and the immune response [16–18]. The spleen is the largest secondary immune organ and comprises two principal compartments: red pulp, which filters the blood of foreign material and damaged and weakened erythrocytes, and white pulp, which initiates immune reactions to blood-borne antigens. For example, the CRH-overexpressing transgenic mouse has high circulating levels of corticosterone, undergoes significant changes in leukocyte populations, and fails to form germinal centers in the white pulp of the spleen [19]. Chronic and exogenous glucocorticoid treatment has the same effect on the immune response in rats [20]. Similarly, long-term administration of synthetic glucocorticoids causes immunosuppression, increasing the risk of infection [21].

We noted alterations in red and white pulps with the various treatments (IMM, HC, and IMM + HC). Restraint stress can cause the spleen to involute [22], impairing the host's ability to mount an immune response [23]; in our experiments, the follicles at white pulp differed in size in the spleen of IMM rats.

*In vitro* and *in vivo*, high corticosterone levels attenuate splenic T-cell proliferation [24]. WKY rats that were treated with hydrocortisone and restraint/hydrocortisone showed significant alterations in immune function, manifesting as greater red pulp and decreased white pulp. This phenomenon might be attributed to desensitization of glucocorticoid receptors in T and B cells due to long-term exposure to elevated corticosterone levels *in vivo*, impairing cell proliferation [24].

As discussed, chronic administration of corticosterone in rats effects behavioral and neurobiological alterations that mirror several of the core symptoms and neurobiological changes that are associated with psychiatric illnesses. Clinical studies have shown that major depressive patients express a pattern of anti-inflammatory cytokines, in association with high cortisol levels [9, 10], which might reflect immunosuppression. In this study, we aimed at stimulating various chronic stress conditions of depressed patients in WKY rats (overactivation of the HPA axis plus administration of chronic stressors) and evaluating the morphological changes in the spleen to understand the mechanisms that underlie the predisposition of depressed patients to disease and infection. Although this study is limited because of sample size (*n* = 6), studies based on larger samples are necessary in order to corroborate these conclusions. Despite of this result, several experiments are needed to elucidate the clinical significance of this finding.

## 5. Conclusion

Based on the function of the spleen as a site of antigen presentation, B- and T-lymphocyte activation, and polarization of cytokine responses, chronic stress induces morphological changes that might alter cellular and humoral immune responses.
